# Deregulated lncRNA expression profile in the mouse lung adenocarcinomas with KRAS‐G12D mutation and P53 knockout

**DOI:** 10.1111/jcmm.14584

**Published:** 2019-08-14

**Authors:** Meiqin Zhang, Nan Jiang, Renjie Cui, Sichen Du, Huayuan Ou, Tinglan Chen, Runsheng Ge, Duan Ma, Jin Zhang

**Affiliations:** ^1^ Key Laboratory of Metabolism and Molecular Medicine, Ministry of Education, Department of Biochemistry and Molecular Biology, School of Basic Medical Sciences, & Institutes of Biomedical Sciences, Shanghai Medical College Fudan University Shanghai China; ^2^ Zhongshan Hospital Fudan University Shanghai China; ^3^ Children's Hospital Fudan University Shanghai China

**Keywords:** hypoxia, *Kras*, long non‐coding RNAs, mouse lung adenocarcinomas, *p53*

## Abstract

Recent studies have demonstrated that aberrant long non‐coding RNAs (lncRNAs) expression are suggested to be closely associated with multiple human diseases, lung cancer included. However, the roles of lncRNAs in lung cancer are not well understood. In this study, we used microarrays to investigate the aberrantly expressed lncRNAs in the mouse lung adenocarcinoma with P53 knockout and the Kras^G12D^ mutation. Results revealed that 6424 lncRNAs were differentially expressed (≥ 2‐fold change, *P* < .05). Two hundred and ten lncRNAs showed more than 8‐fold change and conserved across human and were further analysed in the primary mouse lung adenocarcinoma KP cells, which were isolated from the *p53* knockout and the Kras^G12D^ mutation mice. Among all the 210 lncRNAs, 11 lncRNAs' expression was regulated by P53, 33 lncRNAs by KRAS and 13 lncRNAs by hypoxia in the primary KP cells, respectively. NONMMUT015812, which was remarkably up‐regulated in the mouse lung adenocarcinoma and negatively regulated by the P53 re‐expression, was detected to analyse its cellular function. Results showed that knockdown of NONMMUT015812 by shRNAs decreased proliferation and migration abilities of KP cells. Among those aberrantly expressed lncRNAs in the mouse lung adenocarcinoma, NONMMUT015812 was a potential oncogene.

## 1 INTRODUCTION

As the most prevalent primary malignant tumour in the world, lung cancer has been the leading cause of cancer‐related death.^1^ Non‐small cell lung cancer (NSCLC) is the most common type of lung cancer and includes adenocarcinoma, squamous cell carcinoma and large cell carcinoma.^2^ Adenocarcinoma is the most common type of NSCLC, now accounting for about 40% of all lung cancer cases.^3^ Genetic alterations in non‐small cell lung cancer (NSCLC) have been identified in recent years. *Kristen rat sarcoma* viral oncogene (*KRAS*), *epidermal growth factor receptor* (*EGFR*) and *anaplastic lymphoma kinase* (*ALK*) are the most commonly altered oncogenes by acting as tumour genomic drivers.^4^ More recently, the identification of activating epidermal growth factor receptor (*EGFR)* mutations and anaplastic lymphoma kinase (*ALK*) rearrangements as predictive biomarkers for treatment of NSCLC led to further personalization of therapy with EGFR tyrosine kinase inhibitors (EGFR‐TKIs) and ALK inhibitors. KRAS is mutated to an activated form in ∼30% of NSCLCs; however, effective clinical therapies targeting *KRAS* have yet to be developed. The study of the underlying biology of KRAS in patients with NSCLC could help to determine potential candidates to evaluate novel targeted agents and combinations that may allow a tailored treatment for these patients.^5^ In addition, the *P53* tumour suppressor gene is mutated or deleted in ∼50% human cancers.^6^ Wild‐type (WT) P53 helps maintain genome integrity and cellular homeostasis by regulating the expression of a plethora of genes involved in the regulation of cell cycle, apoptosis, stem cell differentiation, senescence, DNA repair and metabolism.^7^

Long non‐coding RNAs (lncRNAs), a kind of transcript RNA molecular longer than 200 nucleotides, are not translated into protein in the nucleus or cytoplasm. Studies have shown that lncRNAs play significant roles in many life activities such as epigenetic regulation, cell cycle regulation and cell differentiation regulation, and abnormally express in a variety of cancers. The aberrant expression of lncRNAs can be used as tumour promoters or inhibitors. Studies have indicated that lncRNAs play critical roles in lung cancer development and progression by altering multiple signalling pathways, and some cancer‐associated lncRNAs have been identified, such as H19,^8^ MALAT(lung adenocarcinoma associated transcript 1),^9^, ^10^ MIR31HG,^11^ HOTAIR,^12^ DGCR5,^13^ AFAP1‐AS1,^14^ SNHG1 and RMRP.^15^ However, the research on the identification of additional lung cancer‐associated lncRNAs remains to be investigated.

The widely used lung adenocarcinoma mouse model with *p53* deletion and Kras^G12D^ mutation was chosen in this study. The expression patterns of lncRNAs in the mouse lung adenocarcinoma tissue were detected by using microarrays. Based on the microarray results, 210 conserved lncRNAs were selected to detect the relationship between their expression and P53 or KRAS. Besides, hypoxia is an important tumour microenvironmental factor that can induce and even aggravate metastasis,^16^ so we also detected the effects of hypoxia on the 210 lncRNAs. Furthermore, the cellular function of NONMMUT015812 was further investigated in the primary mouse lung adenocarcinoma cells.

## 2 MATERIALS AND METHODS

### 2.1 Mouse tissue samples

The Institutional Animal Care and Use Committee of Fudan University, China, approved all protocols. p53(flox/flox) mice (The Jackson Laboratory) were crossed with the LSL‐Kras G12D mice (The Jackson Laboratory) to generate p53(flox/flox); LSL‐Kras‐G12D mice and the genotyping primers were listed in the Table S1. As described by DuPage,^17^ lung adenocarcinomas were initiated by intranasal instillation with 1 × 10^6^ lentiviral particles expressing Cre‐recombinase per 8‐week‐old mouse. The lung adenocarcinoma samples and corresponding normal lung samples were prospectively collected 6 weeks after tumour initiation under a dissection microscope (Table S2). The tumour size ranges from 2 to 4 mm and, consistent with DuPage's description, these tumours were classified as Grade 2. No obvious metastatic lesions were found in livers and brains.

### 2.2 Microarray and computation

For microarray analysis, lncRNA + mRNA mouse Gene Expression Microarray v1.0, 4x180K chip (CapitalBio Corp), was employed and conducted by CapitalBio Corp according to the manufacturer's protocols. Briefly, 1 μg of total RNA extracted from the samples was transcribed into double‐stranded cDNA using CbcScript reverse transcriptase and T7‐random primer according to the manufacturer's protocol (CapitalBio). Double‐stranded cDNA products were purified and in vitro transcribed to cRNA with T7 RNA polymerase. After reverse transcription, the cRNA was transcribed into cDNA and labelled with Cy3 by using the Klenow enzyme‐labelling strategy, and microarray slide hybridized with the Cy3 labelled probes. Feature Extraction Software v10.7 was used for data extraction from raw microarray images files. Quantile normalization and subsequent data processing were performed using the GeneSpring GX v13.0 software package (Agilent Technologies).

### 2.3 RNA extraction and quantitative real‐time PCR

Total RNA was extracted from cultured cells or tissues using TRIzol reagent (Thermo Fisher) according to the manufacturer's protocol. RNA degradation and contamination were assessed on 1% agarose gels, and RNA concentration was measured using a NanoDrop 1000 (Thermo Scientific). cDNA was synthesized using PrimeScript RT reagent Kit with gDNA Eraser (Perfect Real Time) (TaKaRa), and the integrity of synthesized cDNA was confirmed using glyceraldehyde 3‐phosphate dehydrogenase (Gapdh) or 18s as the endogenous control. Real‐time PCR was carried out using SYBR Premix Ex TaqTM II (Perfect Real Time) (TaKaRa) and measured using ABI 7900 or ABI 7500 instrument. The primers are listed in Table S3. PCR was performed in a total reaction volume of 15 μL, including 7.5 μL of SYBR Premix (2×), 1 μL of cDNA template, 0.75 μL of PCR forward primer (10 mmol/L), 0.75 μL of PCR reverse primer (10 mmol/L), 0.3 μL of ROX Reference (10 mmol/L) and 4.7 μL of double‐distilled water. The qPCR reaction included an initial denaturation step of 30 seconds at 95^ο^C; 40 cycles of 5 seconds at 95^ο^C, 30 seconds at 60^ο^C; and a final melting curve generation programme: 15 seconds at 95^ο^C, 1 minute at 60^ο^C and 15 seconds at 95^ο^C. All experiments were carried out in triplicate or duplicate.

### 2.4 Plasmid expression constructs

*p53* (NM_00546) was generated by PCR and was subcloned into the pCDH‐CMV‐MCS‐EF‐puro lentivirus expression vector (System Biosciences) with FLAG‐tag. The anti‐NONMMUT015812 shRNA was designed by using the InvivoGen's online siRNA Wizard software and subcloned between the *BamHI* and *EcoRI* sites of the pGreenPuro shRNA Expression Lenti‐vector (System Biosciences). The *Kras* CRISPR guide RNA sequences were designed by using the web tool developed by Feng Zhang group at MIT and subcloned between the two *BsmBI* sites of the Lenti‐Guide‐coGFP‐P2A‐Puro guide RNA Expression Lenti‐vector. Plasmid lentiCas9‐Blast from Dr Feng Zhang's laboratory was used to package Cas‐expression lentivirus, and PCDH‐Cre was used to package Cre‐expression lentivirus. All the used DNA oligos were listed in the Table S1.

### 2.5 Cell culture, transfection, lentivirus packaging and infection, and subcutaneous tumour formation assay

HEK293T cells were grown in DMEM supplemented with 10% foetal bovine serum (FBS), 100 U/mL penicillin and 100 mg/mL streptomycin at 37°C with 5% CO_2_ and transfected using Lipofectamine 2000 (Invitrogen) as described in the manufacturer's protocol. Primary murine lung adenocarcinoma cells (KP cells) were isolated from the lung adenocarcinoma tissues of p53(flox/flox); LSL‐Kras‐G12D mice and cultured in RPMI‐1640 containing 10% FBS. To detect the tumour formation ability of the primary KP cells in vivo, 5 × 10^5^ KP cells in 100 μL PBS buffer were subcutaneously injected into the right armpit of 8‐week‐old male C57 mice. After 4 weeks, mice were killed and tumours were dissected for H&E staining. Dissected tumours were fixed with 10% neutral buffered formalin, embedded in paraffin wax, sectioned at a thickness of 5 μm in the coronal plane and stained with haematoxylin and eosin (H&E). As described previously, we used the pPACKH1™ HIV Lentivector Packaging Kit to package pseudoviral particles of the lentiviral shRNA, guider RNA, or gene expression constructs in HEK293T according to the manufacturer's instructions (LV500A‐1, Systems Biosciences). The virus‐containing supernatant was collected, filtered (with 0.45 µm syringe filters) and used to infect the cells in complete media containing 5 µg/mL polybrene (Sigma) after determining the titre of the lentiviral particles. The infected stable KP cells were selected with 4 µg/mL puromycin dihydrochloride (Amresco) or 10 µg/mL blasticidin (Invitrogen) after 72 hours by using lentiviral infection.

### 2.6 Western blot analyses

Treated cells or tissues were lysed by a lysis buffer (200 mmol/L Tris‐HCl (pH 7.5), 1.5 mol/L NaCl, 10 mmol/L EDTA, 10 mmol/L EGTA, 25 mmol/L sodium pyrophosphate, 10 mM β‐glycerophosphate, 1 mmol/L Na_3_VO_4_ and 50 mmol/L NaF) supplied with a protease inhibitor cocktail (Roche Diagnostics). Protein lysates were quantified with Pierce™ BCA Protein Assay Kit (Thermo Fisher). After SDS‐PAGE resolution and membrane transfer, the target proteins were probed with primary antibody against P53 Protein (Gene Tech), HIF‐1α (NOVUS), GAPDH (Bioworld) or β‐tublin (Proteintech) followed by incubation with HRP‐conjugated secondary antibodies. Finally, the bands were visualized using Luminescent Image Analyzer (GE, ImageQuant LAS 4000 mini).

### 2.7 In vitro analysis of cell proliferation, cell cycle, apoptosis and migration

Cells were seeded in 12‐well‐flat‐bottomed plate, with each well containing approximately 1000 cells in 900 μL of cell suspension. After a week, the cells in each well were washed twice by PBS, fixed in 100% methanol for 5 minutes at room temperature, stained with 0.1% crystal violet for 5 minutes, washed four times by PBS and finally air‐dried. For cell cycle analysis, cells were collected and fixed in 70% ethanol overnight at 4°C. Single‐cell suspensions were labelled with PI/RNase Staining Buffer (BD Pharmingen™) and analysed by flow cytometry (Beckman Coulter). Each test (1 × 10^6^ cells) was incubated with 0.5 mL buffer for 15 minutes at room temperature before analysis. For detection of apoptosis, cells were stained with Annexin V and propidium iodide using the Annexin V, 633 kit (DojinDo) according to the manufacturer's protocol, and the percentage of apoptotic cells was determined by flow cytometry (Beckman Coulter).

For migration assay, the transwell inserts were placed in a 24‐well plate with about 1 × 10^4^ cells in serum‐free DMEM media per insert. The inserts were incubated at 37°C for 12 hours in wells containing DMEM media supplemented with 10% FBS. After 12 hours, the inserts were washed with PBS, fixed with methanol for 2 minutes and stained with 0.1% crystal violet for 30 minutes. The cells on the apical side of each insert were then scraped off with a cotton swab. The number of cells that had migrated to the basal side of the membrane was visualized with a microscope at 200× magnification.

### 2.8 RNA sequencing

The NONMMUT015812‐knockdown KP (shRNA‐2) cells and negative control (sh‐Scr) cells were obtained on day 6 of a viral infection. In‐depth RNA sequencing was performed by the Biomarker Technologies in China. The raw sequencing image data were examined via Illumina analysis pipeline and aligned with the unmasked mouse reference genome. Differential expression analysis of two groups was performed using the DESeq R package (1.10.1). DESeq provide statistical routines for determining differential expression in digital gene expression data using a model based on the negative binomial distribution. The resulting *P* values were adjusted using the Benjamini and Hochberg's approach for controlling the false discovery rate. Genes with an adjusted *P*‐value < .01 and absolute value of log2(Fold change)>1 found by DESeq were assigned as differentially expressed.

### 2.9 Construction of lncRNA‐lncRNA co‐expression network and statistical analyses

Based on the computational methods used by Pan JQ,^18^ the expression values of differential expressed lncRNAs (DE lncRNAs) were obtained from Table S4. The correlation coefficient (CC) and significant *P*‐value were calculated between the expression values of each lncRNA‐lncRNA pair across all samples. The lncRNA‐lncRNA pairs with a *P*‐value < .001 and absolute value of CC > 0.9 were used to construct a lncRNA‐lncRNA co‐expression network (LLCN). The LLCN was generated by using Cytoscape software.

Statistical differences (*P*‐values) among groups were obtained using two‐sided Student's *t* test. Results were considered to be significant for *P* values under .05. All statistical tests were performed using Prism software (GraphPad, version 5.0).

## 3 RESULT

### 3.1 LncRNA expression profile in the mouse lung adenocarcinomas with KRAS‐G12D mutation and *p53* knockout

**Figure 1:**
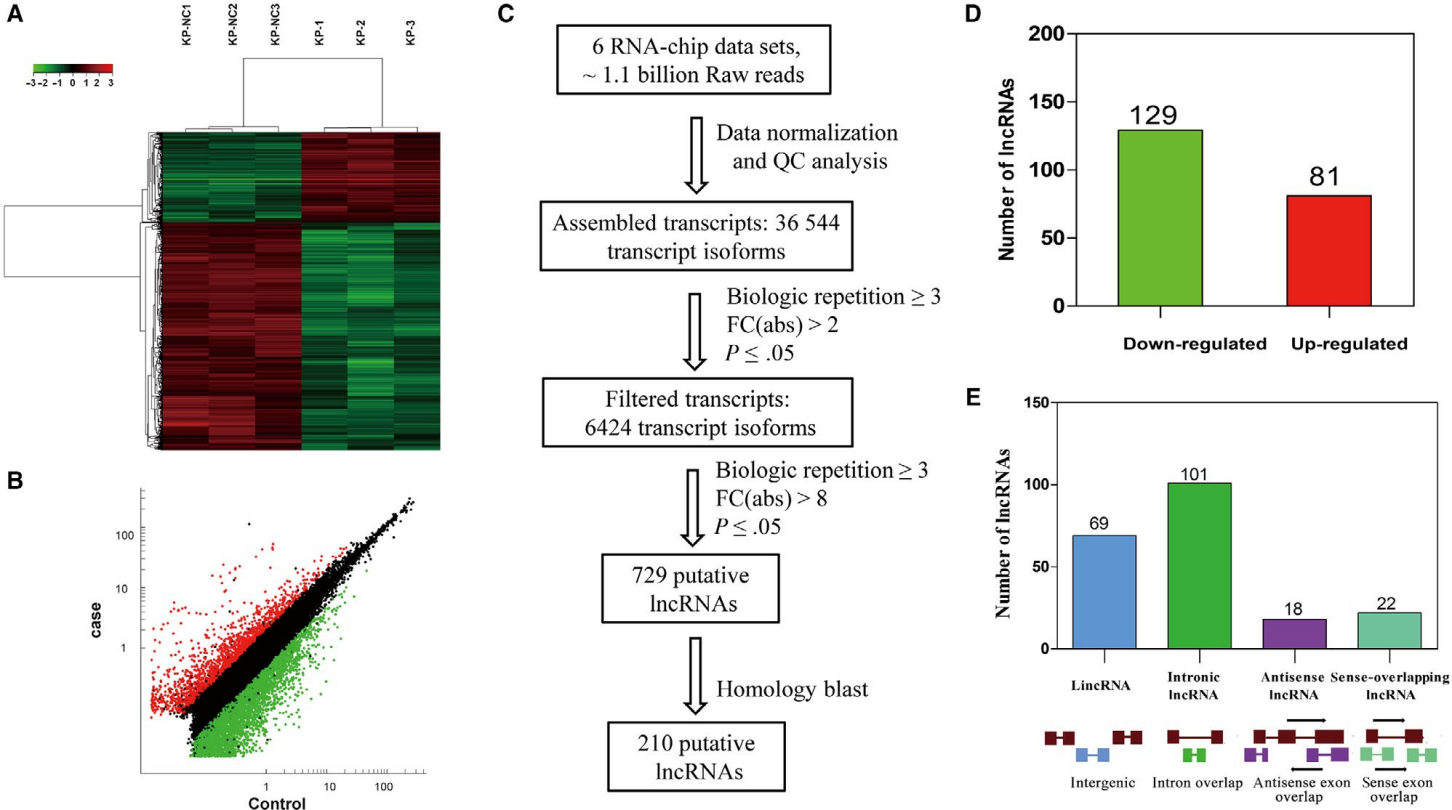
The variation in lncRNA expression between the lung adenocarcinoma and normal lung tissue arrays. A, Heat map and hierarchical clustering of lncRNA profile comparison between the lung adenocarcinoma and normal lung samples of three p53(flox/flox);LSL‐Kras‐G12D mice. B, Scatter plot showing the variation in lncRNA expression between the lung adenocarcinoma and normal lung tissue arrays. The values of the X and Y in the scatter plot are signal values. Red dots and green dots represent up‐regulated genes and down‐regulated genes, respectively. Black bots mean those genes with no difference in lncRNA expression between case and control. C, Flowchart showing the selection process for the 210 conserved lncRNAs with expression change greater than 8‐fold. D, The selected 210 lncRNAs contains 129 down‐regulated and 81 up‐regulated ones. E, Classification of the 210 selected lncRNAs, red rectangles or lines represent the exon or intron of protein‐coding gene, respectively; Blue, green, purple and light blue rectangles or lines represent the exon or intron of lncRNA, respectively

As described by DuPage, nasal inhalation with 1 × 10^6^ Cre‐expressing lentiviral particles per mouse was carried out to induce lung adenocarcinoma formation in p53(flox/flox); LSL‐Kras‐G12D C57 mice by activating Kras‐G12D expression and P53 deletion and the lung adenocarcinoma tissues were collected to detect the lncRNA expression profiles through microarray analysis containing probes for 58 809 mouse lncRNAs, with the normal mouse lung tissues as controls. Of the detectable lncRNAs, analysis of the normalized data revealed 6424 differentially expressed lncRNAs（≥ 2‐fold change, *P* < .05）, including 1821 up‐regulated and 4602 down‐regulated. Scatter plot and hierarchical clustering, based on differentially expressed lncRNAs, showed clear distinction between lung adenocarcinoma and normal lung samples (Table S4 and S5, Figure 1A and 1B). We focused on the lncRNAs which exhibited higher fold changes and were conserved between human and mouse. When the threshold (fold change > 8, *P* < .05) was set, 729 lncRNAs were retained. Furthermore, based on positional conservation in chromosome and sequence conservation analysed by the web Blastn program at the National Center for Biotechnology Information (NCBI), 210 lncRNAs with potential human orthologs were screened for follow‐up verification, including 129 up‐regulated and 81 down‐regulated (Figure 1C and 1D). The 210 lncRNAs can be classified into four categories including ‘u' (intergenic), ‘i' (intronic), ‘x' (anti‐sense) and ‘o' (sense overlapping) according to their genomic location and referring to the neighbouring genes.^19^ Most of the identified lncRNAs fell into class i, with 101 lncRNAs (48.09%), whereas 69 (32.86%), 18 (8.57%) and 22 (10.48%) lncRNAs belonged to classes i, x and o, respectively (Figure 1E).

### 3.2 Isolation and identification of KP cell

**Figure 2:**
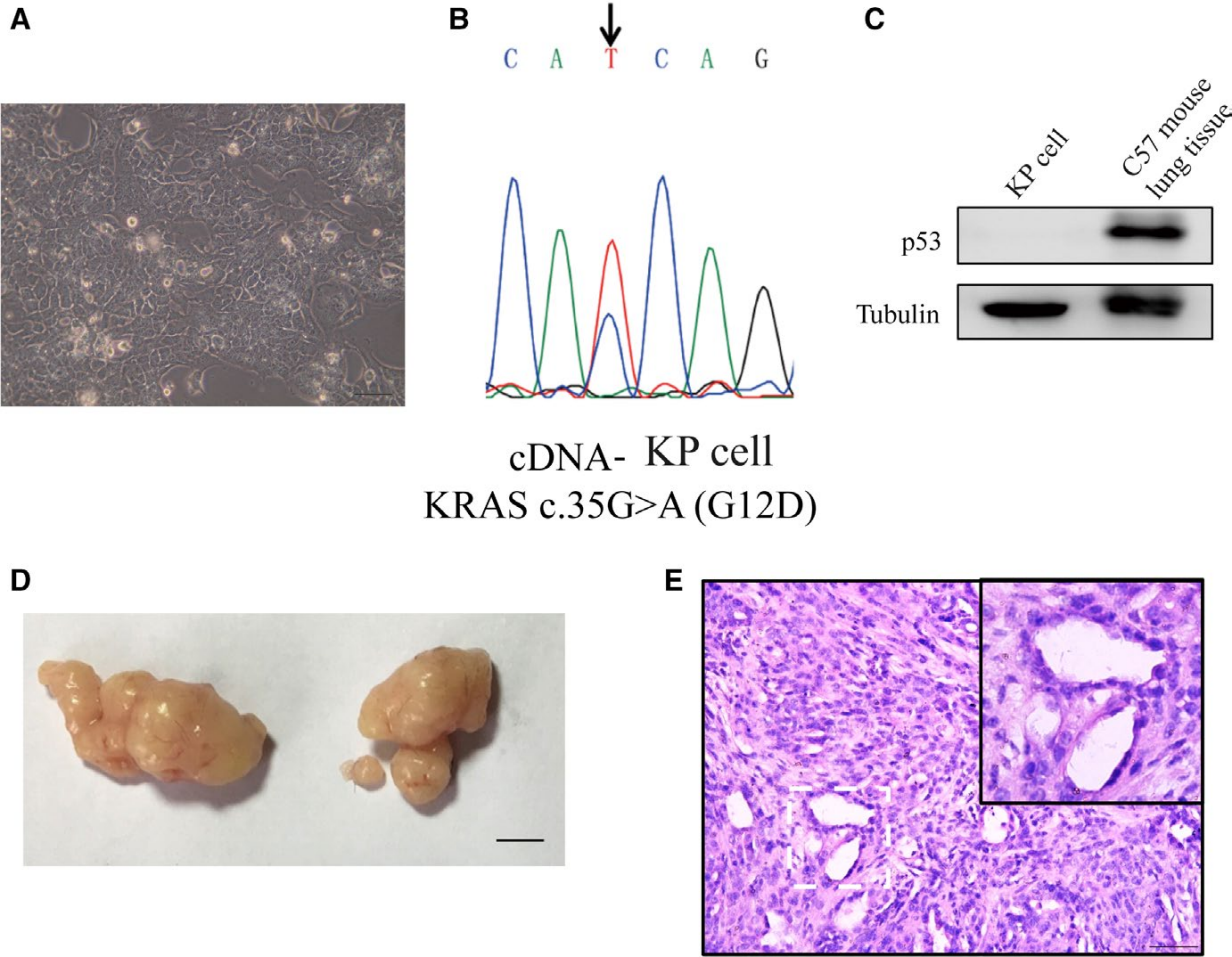
Isolation and identification of KP cells. A, Micrograph of KP cells. Scale bars, 0.1mm. B, The DNA sequencing of the products of *Kras* gene cDNA RT‐PCR showed that *Kras* G12D expressed in the isolated KP cells. C, P53 was deleted in the KP cells. Western blot analysis was conducted in the KP cells and the normal lung tissue from LSL‐Kras^G12D^; *p53^flox/flox^* C57 mouse was used as positive control. D, Representative images of tumour at 4 weeks after subcutaneous injection of KP cells in C57 mice. Scale bars, 5mm. E, These formed tumours were confirmed by Haematoxylin and eosin (H&E) staining. Scale bars, 0.1 mm

In order to identify the possible factors deregulating lncRNAs expression of the mouse lung adenocarcinoma in vitro, primary lung adenocarcinoma cells with K‐ras^G12D^;p53^−/−^, namely KP cell in our research, were isolated from the lung adenocarcinoma tissues of p53(flox/flox);LSL‐Kras‐G12D C57 mice induced by nasal inhalation of lentivirus‐Cre. The primary KP cells could stably grow and continuously passage, most of them appeared irregular polygon, and they could grew in overlap and pile up in high density (Figure 2A). The *Kras* mutation gene (c.35G > A) was confirmed by DNA sequencing (Figure 2B). Western blot showed that P53 was completely deleted in the KP cells (Figure 2C). The primary KP cells derived from p53(flox/flox);LSL‐Kras‐G12D C57 mice, so they could theoretically form tumour in wild‐type C57. To assess the capability of tumour formation, KP cells were subcutaneously injected into armpits of wild‐type C57 mice, and obvious tumour formed within one month and adenocarcinoma was confirmed by the H&E staining analysis (Figure 2D and 2E).

### 3.3 Analysis of lncRNAs associated with *p53 Kras* or hypoxia

**Figure 3:**
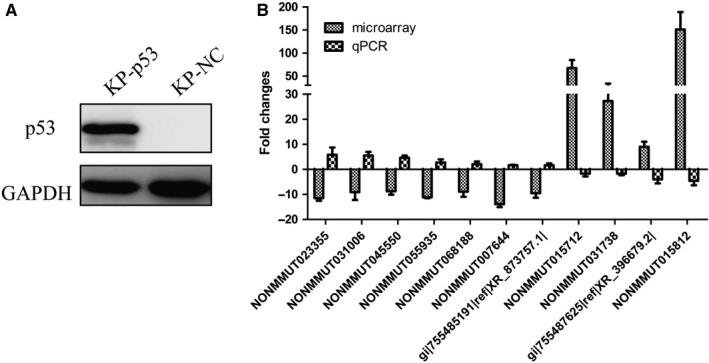
Construction of P53 re‐expression KP cells and identification of lncRNAs regulated by P53. A, P53 was efficiently re‐expressed in the primary KP cells. The P53‐re‐expressed KP cells (KP‐P53) were developed by PCDH‐Flag‐P53 lentiviral infection and puromycin selection （4 μg/mL）, using PCDH‐Flag empty vector lentivirus as negative control. Western blot analysis was used to analyse the expression of P53 in lysates from KP cells. B, Comparison between microarray data and qPCR results of lncRNAs regulated by P53. Among the selected 210 lncRNAs, qPCR results determined 11 lncRNAs, whose expression was altered by the re‐expression of P53 in KP cells and exhibited opposite expression alteration direction to that in the microarray of the mouse lung adenocarcinoma tissues. The heights of the columns in the chart represent the log‐transformed median fold changes (tumour sample/normal tissues in microarray data or KP‐*p53* cells/KP‐NC cells in qPCR results). Mean ± SD are shown, n = 3 or 2

*p53* is the most common tumour suppressor gene mutated in clinical lung cancer, and it was induced to complete knockout in our lung adenocarcinoma mouse model and the KP cells. Thus, we explored whether the deregulated expression of the selected conserved 210 lncRNAs was associated with the *p53* deletion. P53 was re‐expressed in the KP cells by the lentiviral particles carrying the *p53* gene. As shown in Figure 3A, P53 was efficiently re‐expressed in the stably infected KP cells. The quantitative PCR assay showed that expression of 11 lncRNAs from the selected 210 lncRNAs was significantly altered by P53 re‐expression (fold change ≥ 1.5), and the fold change was contrary to the above microarray data of lung adenocarcinoma tissues in which *p53* was deleted (Figure 3B).

**Figure 4:**
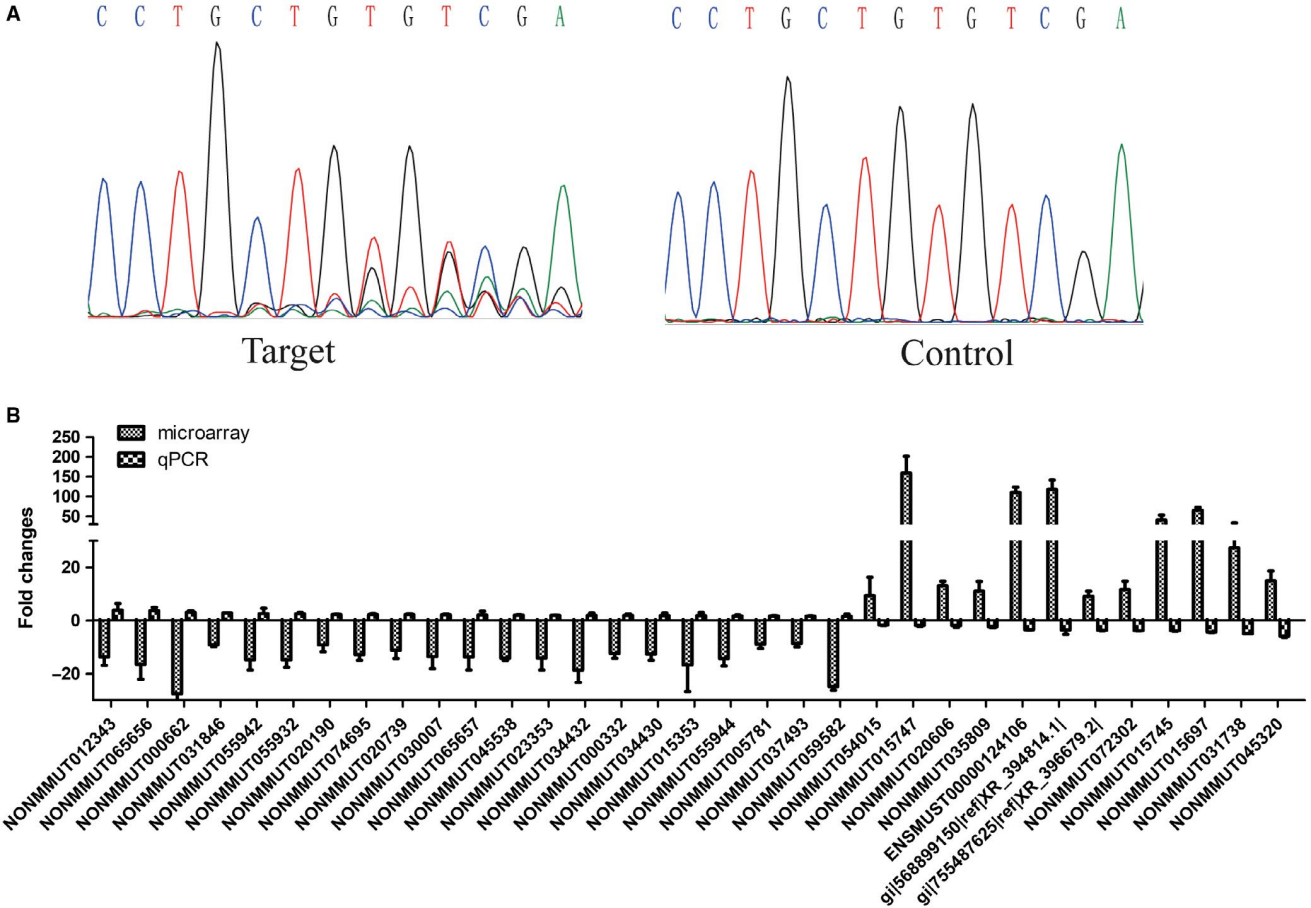
Construction of KP cell lines with *Kras gene* disruption and identification of *Kras* related lncRNAs in KP cells. A, Messy peak figure reporting the results of DNA sequencing showed mutations induced by Cas9/gRNA in the *Kras* guide RNA. The stable Cas9 expression KP cells (KP‐Cas9) were developed by pCDH‐bla‐Cas9 lentivirus infection and selection with 10 μg/mL blasticidin. KP‐Cas9 cells were further infected by the *Kras*‐targeting guide RNA expression lentivirus and selected by 4 μg/mL puromycin, lenti‐guide empty vector lentivirus as negative control. The DNA fragment containing the Kras guide RNA binding site was amplified by using PCR with the genomic DNA of guide RNA‐infected KP‐cas9 cells. B, Comparison between microarray data and qPCR results of lncRNAs regulated by *Kras*. Among the selected 210 lncRNAs, qPCR results determined 33 lncRNAs, whose expression was altered by the *Kras* deletion in KP cells and exhibited opposite expression alteration direction to that in the microarray of the mouse lung adenocarcinoma tissues. The heights of the columns in the chart represent the log‐transformed median fold changes (tumour sample/normal tissues in microarray data of the mouse lung adenocarcinoma tissues or KP‐Kras‐guide cells/ KP‐guide‐NC cells in qPCR results). Mean ± SD are shown, n = 3 or 2

*Kras* was activated in the lung adenocarcinoma mouse model and the KP cells. Similarly, to identify lncRNAs whose expression was relevant to the *Kras* status, we used the Crispr/Cas9 targeting the coding sequence region of *Kras* gene to knockout its expression in KP cells. The DNA sequencing of genomic PCR products containing the guider RNA binding site showed that the *Kras* gene was efficiently mutated in KP cells by the Crispr/Cas9, and the cell growth was obviously inhibited (Figure 4A). The quantitative PCR assay identified 33 lncRNAs whose expression was affected by the deletion of *Kras* (fold change ≥ 1.5), and the fold change was also contrary to the original microarray data of lung adenocarcinoma tissues in which *Kras* was activated (Figure 4B).

**Figure 5:**
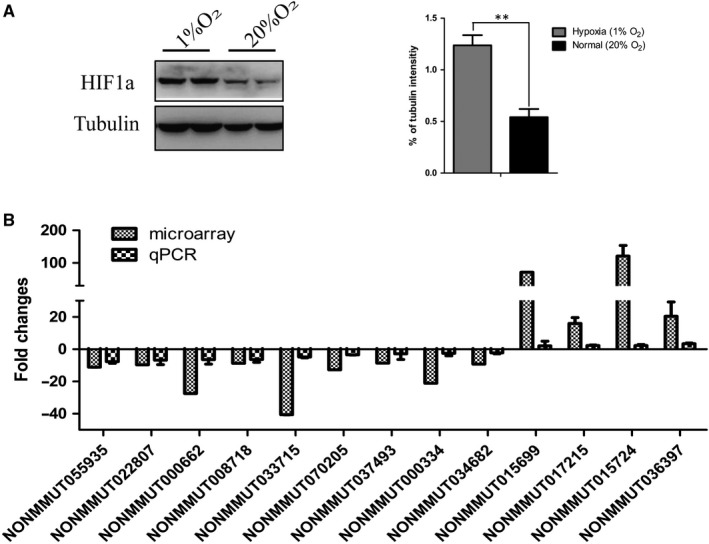
Identification of hypoxia‐related lncRNAs in KP cells. A, KP cells were incubated under hypoxia with 1% O_2_ and normoxia (20% O_2_). Immunoblot (left) was used to detect HIF1α protein levels and was quantified by measuring the grey value of the bands. Data are shown as means ± SD. All *P* values are from two‐sided *t* tests. B, Comparison between microarray data and qPCR results of lncRNAs associated with hypoxia. The qPCR result validated the 13 out of 210 selected lnRNAs were regulated by hypoxia, whose expression exhibited same expression alteration direction to that in the microarray of the mouse lung adenocarcinoma tissues. The heights of the columns in the chart represent the log‐transformed median fold changes (tumour sample/normal tissues in microarray data of the mouse lung adenocarcinoma tissues or KP cells cultured in hypoxia/KP cells cultured in normoxia in qPCR results) in expression across the three lung adenocarcinoma tissues and KP cells cultured in hypoxia. Mean ± SD are shown, n = 3 or 2

Furthermore, hypoxia or oxygen deficiency is a prominent feature of solid tumours. Hypoxic tumours are often resistant to conventional cancer therapies, and tumour hypoxia correlates with advanced stages of malignancy and metastasis.^20^, ^21^, ^22^ We further investigated the potential lncRNAs regulated by hypoxia. KP cells were cultured in normoxia (20% O_2_) vs hypoxia (1% O_2_) for up to 48 hours, and Western blotting showed that hypoxia effectively elevated the expression of HIFα in KP cells (Figure 5A). Expression of the selected 210 lncRNAs was quantified by real‐time PCR in the hypoxia‐induced KP cells and the results showed that 13 lncRNAs were down‐regulated, and four lncRNAs were up‐regulated by hypoxia, which exhibited the same expression alteration direction to that in the microarray of with the mouse lung adenocarcinoma tissues (Figure 5B).

**Figure 6:**
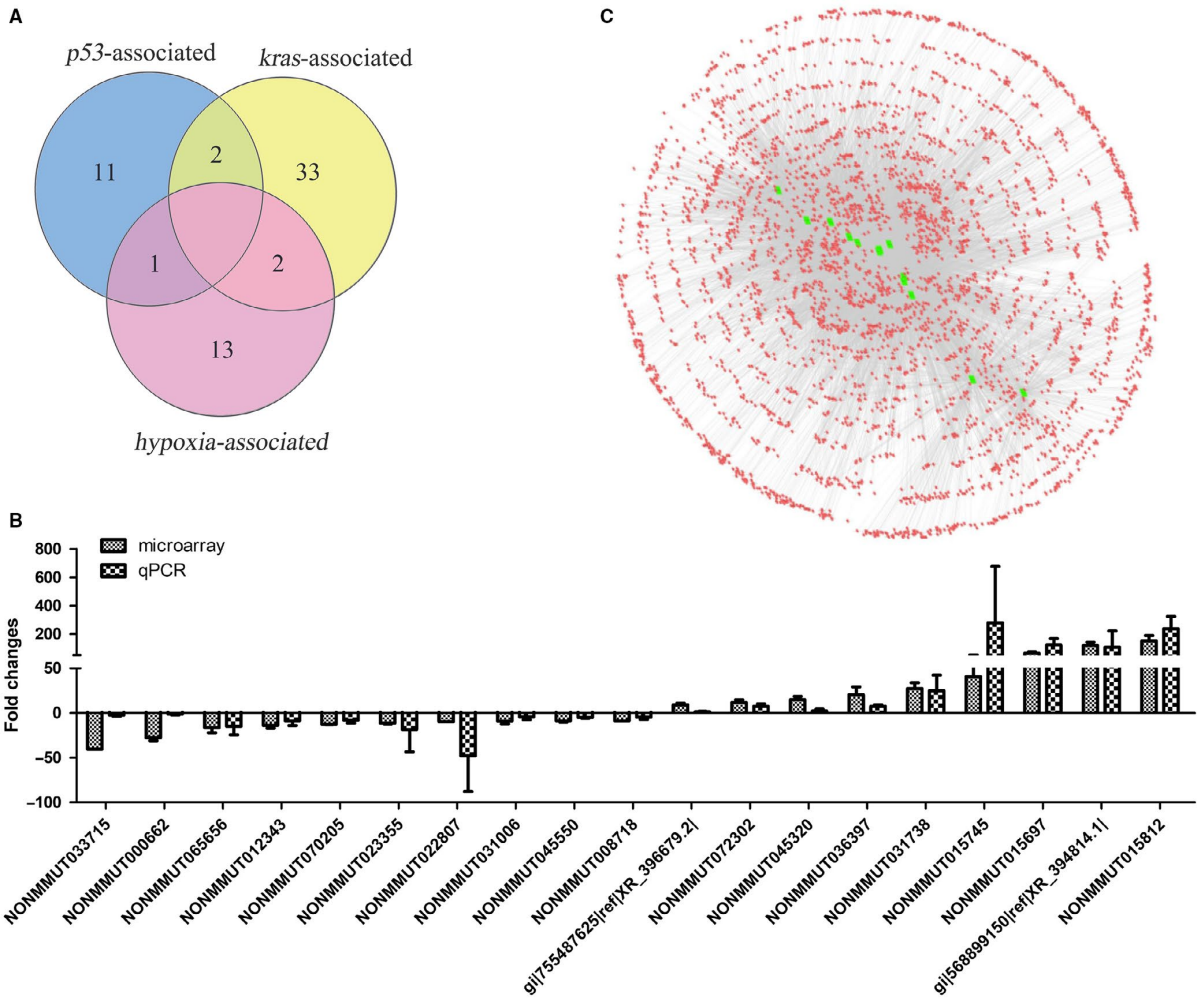
Confirmation of the *p53‐*, *Kras‐* or hypoxia‐ associated lncRNAs. A, Overlapped differentially expressed lncRNAs. B, Among the total 52 lncRNAs identified to be associated with *p53*, *Kras* or hypoxia, 19 lncRNAs, with expression change fold greater than 3.5 in the microarray results, were selected to further confirmed by using quantitative RT‐PCR in another group of mouse lung adenocarcinoma tissues (6 lung adenocarcinoma samples and 6 normal lung tissues). The heights of the columns in the chart represent the log‐transformed median fold changes (tumour sample/normal tissues). The validation results of the 19 lncRNAs indicated that the microarray data correlated well with the qPCR results. Mean ± SD are shown, n = 3 or 6. C, Long noncoding RNA (lncRNA)‐lncRNA co‐expression network (LLCN) related to the 19 selected lncRNAs was constructed. Among the 19 lncRNAs, 6 lncRNAs had no significant co‐expression with other lncRNAs. The circles represent nodes of differential expressed lncRNAs (DE lncRNAs). The nodes in green colour represent the 13 remained lncRNAs. The LLCN contained a total of 4086 lncRNAs with 19 595 edges between them

Totally, among the 210 selected conserved lncRNAs identified in the microarrays, the expression alteration of 52 lncRNAs showed association with *p53*, *Kras* or hypoxia. Some results further implied that two P53‐regulated lncRNAs (NONMMUT031738, gi|755487625|ref|XR_396679.2|) also were regulated by *Kras*, two *kras*‐regulated lncRNAs (NONMMUT000662, NONMMUT037493) and P53‐regulated lncRNA (NONMMUT055935) could be regulated under hypoxia (Figure 6A). Nineteen lncRNA with the fold change greater than 3.5 were selected to further validate their expression in the mouse lung adenocarcinoma tissues of six lung adenocarcinoma samples and six normal lung tissues by using quantitative RT‐PCR. The quantitative RT‐PCR analysis showed that the expression of 19 lncRNAs was consistent with the microarray results (Figure 6B). The correlation coefficient (CC) and significant *P*‐value were calculated between the expression values of each lncRNA‐lncRNA pair across all samples and were used to construct a lncRNA‐lncRNA co‐expression network (LLCN) related to the above‐identified 19 lncRNAs. Among the 19 lncRNAs, 6 lncRNAs had no significant co‐expression with other lncRNAs. The LLCN contained a total of 4086 lncRNAs with 19 595 edges between them (Figure 6C, Table S6, S7).

### 3.4 Knockdown of NONMMUT015812 suppressed KP cell proliferation and migration

**Figure 7:**
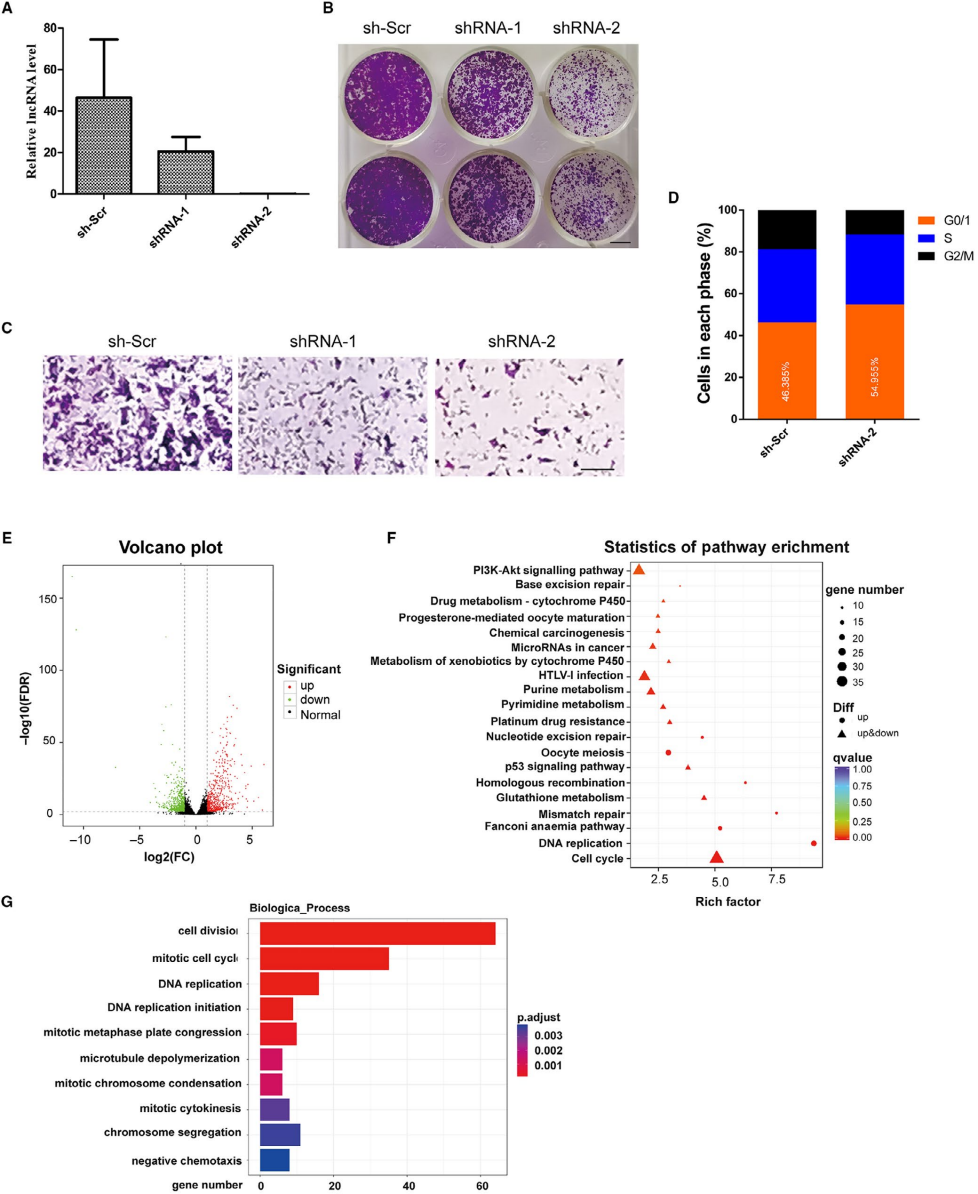
NONMMUT015812 is a potential oncogene. A, NONMMUT015812 was knocked down in the KP cells. Relative expression levels of NONMMUT015812 were determined by qRT–PCR in the KP cells infected with lentivirus of shRNA‐1 and shRNA‐2 to target NONMMUT015812, with the non‐specific shRNA as negative control. Results are shown as means ± SD. B, Knockdown of NONMMUT015812 inhibited colony formation in KP cells. C, Transwell migration assay of the KP cells stably expressing shRNAs. Compared with the non‐specific shRNA as negative control, knockdown of NONMMUT015812 significantly inhibited the cell migration. D, Flow cytometry assays showed that NONMMUT015812 knockdown inhibited the cell cycle progression in KP cells. E, A volcano plot of differentially expressed genes in the NONMMUT015812‐knockdown KP cells. RNA sequencing revealed that a total of 1281 genes were differentially expressed between the NONMMUT015812‐knockdown KP cells and the negative control (up‐regulated: 610, down‐regulated: 671). F, KEGG pathway analysis of the differentially expressed genes in the NONMMUT015812‐knockdown KP cells. G, Gene Ontology analysis of the differentially expressed genes in the NONMMUT015812‐knockdown KP cells, which revealed that many differentially expressed genes were mainly enriched in cell division, mitotic cell cycle and DNA replication processes (*P* < .001)

Among the identified lncRNAs, NONMMUT015812 was very interesting. (a) Its expression increased approximately 150‐ 187‐fold in the mouse lung adenocarcinoma tissues. (b) Its expression was negatively associated with P53. (c) The gene for NONMMUT015812 is located on the chromosome between the genes for retrotransposon‐like protein 1 and thyroxine 5‐deiodinase, in which several differently expressed lncRNAs were included and conserved in human and mouse (this region will be described in detail in the discussion). There were no reports about NONMMUT015812, so we further studied its cellular function in the KP cells. Lentivirus‐mediated shRNAs were designed to knockdown the expression of NONMMUT015812, and the results of quantitative PCR showed that they could reduce NONMMUT015812 in KP cells by more than 80% (Figure 7A). Clone forming assays showed that knockdown of NONMMUT015812 significantly inhibited KP cell growth (Figure 7B). Moreover, in transwell assays, KP cells with stable NONMMUT015812 knockdown showed significantly decreased migration compared with control cells (Figure 7C). Flow cytometry analysis indicated that NONMMUT015812 knockdown resulted in a substantial retardation of KP cells in G0/G1 phase, with decrease in the number of cells in G2/M phase (Figure 7D). However, knockdown of NONMMUT015812 had no significant effects on the apoptosis (Figure S1).

RNA sequencing was used to monitor the change in gene expression in the NONMMUT015812‐knockdown cells. Compared with the negative control, 1281 differentially expressed (DE) genes were identified. Among those genes, 610 genes were up‐regulated, and 671 genes were down‐regulated (Figure 7E, Table S8 and S9). KEGG pathway analysis indicated that down‐regulation of NONMMUT015812 affected several cell growth‐associated pathways, including cell cycle, DNA replication and PI3K/Akt signalling pathway (Figure 7F, Table S10). Interestingly, P53 signalling and several DNA repair pathways were also included in the KEGG pathway enrichment, which means that NONMMUT015812 might be an important downstream mediator of P53 pathway. Consistent with the results of flow cytometry analysis, no apoptosis signalling pathway was identified. Gene Ontology analysis also revealed that many differentially expressed genes were mainly enriched in cell division, mitotic cell cycle and DNA replication processes (Figure 7G, Table S11).

## 4 DISCUSSION

Lung cancer is the leading cause of cancer death, and the majority of cases are diagnosed as NSCLC, in which lung adenocarcinoma is the most common type.^23^ Recently, significant progress has been made in individualizing therapy based on molecular aberrations and pathologic subtype. For example, the use of EGFR–tyrosine kinase inhibitors or *ALK inhibitors* has greatly improved progression‐free survival in NSCL patients with EGFR mutations or ALK translocations. *KRAS* and *P53* are among the most frequently mutated genes in non‐small cell lung cancer, but no targeted therapy has been approved for *KRAS* mutant or *P53* mutant NSCLC at present. Therefore, a better understanding of the pathogenesis of lung adenocarcinoma with *KRAS* or *P53* mutation could provide more effective diagnostic markers or treatment targets for lung adenocarcinoma.

Recent investigations have provided good evidence that lncRNAs play important roles in many biological processes, from chromatin modification, transcription, splicing and translation to cellular differentiation, cell cycle regulation and stem cells reprogramming.^24^ Furthermore, some lncRNAs have been demonstrated to be aberrantly expressed in lung cancer tissues and be associated with cancer progression. For example, HOTAIR is up‐regulated in lung cancer specimens and displayed potential tumour‐promoting roles 25, 26, 27; MALAT1 is a critical regulator of the metastasis phenotype of lung cancer cells, as a competing endogenous RNA (ceRNA) to regulate autophagy‐related 7 (ATG7) gene expression by sponging miR142‐3p.^28^

To investigate lncRNAs deregulated in *p53‐* and *Kras*‐mutated lung cancer, the microarrays were used to analyse lncRNA expression profiles in the tissues of mice lung adenocarcinoma tissues with *p53* gene deletion and *Kras* activation induced by nasal inhalation of Cre‐expressing lentivirus in p53(flox/flox);LSL‐Kras‐G12D C57 mice. A total of differentially expressed 6424 lnRNAs were identified, including 1821 up‐regulated lncRNAs and 4603 down‐regulated in lung adenocarcinoma tissues. Among them, 210 highly dysregulated lncRNAs (fold change ≥ 8) with high human homology were selected for further study (Figure 1). As the mouse lung adenocarcinoma was induced by the P53 knockout and K‐ras^G12D^ expression, it was expected that some of these identified lncRNAs were associated with the status of *p53* and *Kras*. The mouse lung adenocarcinoma cells (KP cells) were isolated from the cancer tissues, and the deletion of P53 and Kras‐G12D mutation was confirmed in the KP cells. With P53 re‐expression or Crispr/Cas9‐mediated *Kras* gene disruption in the KP cells, we identified 11 P53‐associated lncRNAs (Figure 3) and 33 *Kras*‐associated lncRNAs (Figure 4). Hypoxia is a major feature of solid tumours at a status of decreased availability of oxygen, which increases patient treatment resistance and favours tumour progression. Thus, we also investigated the expression of the selected 210 LncRNAs in KP cells under hypoxic condition and the results indicated that expression of 13 lncRNAs was affected by hypoxia. Besides, some lncRNAs showed to be regulated by two factors.

We noticed an intriguing phenomenon that 16 of the identified conserved lncRNAs located on the chromosome region between the genes for retrotransposon‐like protein 1 and thyroxine 5‐deiodinase, they were all up‐regulated in the mouse lung adenocarcinoma tissues, and expression of 9 lncRNAs was identified to be associated with *p53*, *Kras* mutation or hypoxia. The lncRNA maternally expressed 3 (MEG3) reported to be correlated with tumorigenesis,^29^, ^30^ was also included in this region and was identified to be up‐regulated in the mouse lung adenocarcinoma tissues (corresponding to the lncRNA NONMMUT015697) and regulated by *kras* in our results. The above results suggested that this region contain a cluster of tumorigenesis‐promoting lncRNAs. The lncRNA NONMMUT015812, which our results showed to be highly up‐regulated in the mouse lung adenocarcinoma by more than 150‐fold and suppressed by P53, was another lncRNA in this region, and there was no research on its function. Our functional analysis showed that knockdown of NONMMUT015812 suppressed KP cell proliferation and migration, which means it was a candidate oncogene. The RNA‐seq further indicated that NONMMUT015812 mainly regulated cell cycle to control cell growth（Figure 7）.

In conclusion, the deregulated lncRNA expression profile was determined in the mouse lung adenocarcinomas with Kras‐G12D mutation and *p53* knockout. The expression of some highly dysregulated and conserved lncRNAs was revealed to be associated with *p53*, *Kras* or hypoxia, among which the lncRNA NONMMUT015812 was a potential tumorigenesis‐promoting gene. However, the function and molecular mechanism of the identified lncRNAs in tumorigenesis and their expression in human lung cancer tissues need further investigating.

## CONFLICT OF INTEREST

The authors declare that they have no conflicts of interest.

## AUTHOR CONTRIBUTIONS

Jin Zhang and Duan Ma designed the study. Meiqin Zhang performed the experiments and was primarily responsible for writing the manuscript. All the authors contributed to data analysis and manuscript editing and approval.
