# Supplementary figures and images for: Deregulated lncRNA expression profile in the mouse lung adenocarcinomas with KRAS‐G12D mutation and P53 knockout
Source: J Cell Mol Med. 2019 Aug 14;23(10):6978–88. doi: 10.1111/jcmm.14584 (PMC6787463; doi:10.1111/jcmm.14584)

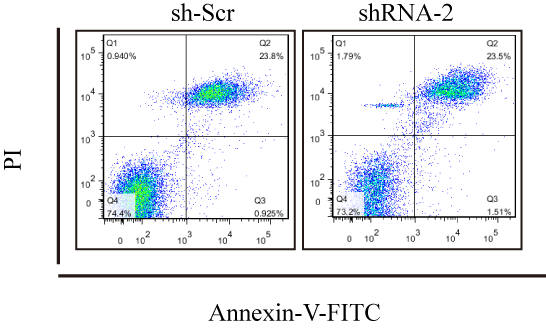

Supplement: Supplementary file 1 [file JCMM-23-6978-s001.tif]
